# Isolation, Biocontrol Efficacy and Genomic Analysis of *Trichoderma harzianum* Snef3255 Against Peanut Root-Knot Nematode Disease

**DOI:** 10.3390/microorganisms14081851

**Published:** 2026-08-20

**Authors:** Xuesong Bai, Zhigang Li, Chenjun Duan, Di Zhao, Hai Dong, Xiaofeng Zhu, Haiyan Fan, Xiaoyu Liu, Yuxi Duan, Lijie Chen

**Affiliations:** 1 Nematology Institute of Northern China, College of Plant Protection, Shenyang Agricultural University, Shenyang 110866, China; 2 Sanya Institute of Breeding and Multiplication/School of Tropical Agriculture and Forestry, Hainan University, Haikou 570228, China; 3 Northeast Germplasm Resources Innovation and Utilization Research Center (Analytical and Testing Center), Shenyang Agricultural University, Shenyang 110866, China; 4 Institute of Plant Protection, Liaoning Academy of Agricultural Sciences, Shenyang 110161, China; 5 College of Science, Shenyang Agricultural University, Shenyang 110866, China

**Keywords:** *Trichoderma harzianum*, peanut, *Meloidogyne hapla*, biocontrol, comparative genomics

## Abstract

Peanut root-knot nematode (RKN) disease caused by *Meloidogyne hapla* is among the most destructive soil-borne diseases threatening peanut production worldwide. We isolated *Trichoderma harzianum* strain Snef3255 from peanut rhizosphere soil, which exhibited high virulence against second-stage juveniles, with a mortality rate of 86.08%. Field trials demonstrated that TC and TS provided control efficacies of 70.85% and 61.88%, respectively, while boosting peanut yields by 18.15% and 13.61%. We report a high-quality chromosome-level genome assembly of *T. harzianum* Snef3255 using Oxford Nanopore, Illumina and high-throughput chromatin conformation capture (Hi-C) sequencing data. This assembly comprised seven chromosomes (40,811,729 bp) with a BUSCO completeness of 99.31%. A total of 13,611 protein-coding genes were predicted. Systematic comparative genomic analysis of *T. harzianum* Snef3255 with other *Trichoderma* species was performed, and putative functional gene clusters were investigated. Furthermore, we identified two candidate secreted proteins, ThCP1 and ThLysM1, and screened their interacting proteins in peanut, respectively. This study demonstrated the potential of Snef3255 as a biocontrol agent for peanut RKN disease and provided a genomic basis for understanding *Trichoderma*–peanut interactions.

## 1. Introduction

Root-knot nematodes (RKNs) are among the most destructive plant-parasitic nematodes worldwide, threatening the growth and yield of more than 3000 plant species, including oilseed and vegetable crops [1,2]. Peanut (*Arachis hypogaea* L.) is susceptible to several root-knot nematode (RKN) species, including *Meloidogyne arenaria*, *M. hapla*, *M. incognita* and *M. javanica* [3,4,5]. Among them, *M. hapla* is a major causal agent of peanut RKN disease, and its effective management remains challenging [6]. Biological control using antagonistic fungi has become an important component of integrated RKN management [7]. Although *Trichoderma* spp. have been widely investigated for their antagonistic activity against RKNs, their biocontrol potential against *M. hapla* in peanut remains insufficiently explored [4,8,9].

*Trichoderma* spp. are well recognized as efficient biocontrol fungi against nematodes [10]. *Trichoderma* species comprise a diverse group of beneficial antagonists that play important roles in promoting plant growth, facilitating nutrient absorption and inducing plant defense responses [10,11]. Among them, *T. harzianum* is considered one of the most important and extensively studied biocontrol species. Its strong biocontrol capacity is mediated through multiple mechanisms, including interspecific resource competition, and the elicitation of systemic defensive signaling in host plant tissues [12,13].

To thoroughly understand the multifaceted beneficial properties of *Trichoderma* species and their potential agricultural applications, a comprehensive elucidation of their physiological characteristics and genetic basis is crucial [10]. High-throughput sequencing platforms have witnessed tremendous advances and widespread adoption over recent years, whole-genome sequencing (WGS) has become a fundamental approach for comprehensively understanding the genus *Trichoderma*. The integration of genomic information with physiological phenotypic data provides an important foundation for the effective development and utilization of fungal strains [14]. In *Trichoderma* species, genome sequence analysis not only offers insights into the biosynthetic machinery responsible for secondary metabolite production, but also reveals key genetic determinants associated with growth, development, and carbohydrate metabolism [15]. Over the past two decades, studies on *Trichoderma* genome sequencing have steadily increased [16,17,18]. Comparative genomic analyses of the most frequently studied *Trichoderma* species provide insights into the evolutionary events underlying nutritional diversification and environmental adaptation [19]. Furthermore, cross-fungal comparisons of the *Trichoderma* core genome with those of other antagonists can help reveal lineage-specific genomic features associated with its industrial and agricultural importance [20].

Genomic-level insights are essential for deciphering the gene clusters in *Trichoderma* that drive the synthesis of bioactive secondary metabolites, including terpenoids, PKS, and NRPS [21,22,23,24,25]. PKS1 drives biosynthesis of the nematicidal volatile 6-pentyl-α-pyrone (6-PP) [23,24], while P450-mediated detoxification boosts soil colonization fitness [25]. These metabolites underpin the efficacy of *Trichoderma* in biocontrol, plant biomass facilitation, and stress mitigation. Moreover, precise genomic information enables the characterization of elicitors, including cell wall-derived polysaccharides and secreted proteins (e.g., Cerato-platanins, LysM), which may act as or modulate microbe-associated molecular patterns (MAMPs) [26,27]. By triggering plant innate immunity and systemic resistance, these elicitors can enhance plant resilience against biotic and abiotic challenges. Nevertheless, chromosome-level *T. harzianum* genome assemblies with seven complete chromosomes and well-curated gene annotations remain scarce in public genomic repositories. This limitation hampers the accurate delineation of biosynthetic gene clusters, secreted proteins such as Cerato-platanins, and secondary metabolite pathways potentially associated with nematicidal activity.

Previously, the RKN species infesting peanut in Liaoning, China was identified as *M. hapla* [28]. However, no *T. harzianum* strain has yet been reported for the biocontrol of *M. hapla* in peanut. In this study, we identified a highly efficient nematicidal *T. harzianum* strain, Snef3255. To further elucidate the genetic basis underlying its biocontrol potential, we conducted chromosome-level genome sequencing, genome annotation, and comparative genomic analyses of Snef3255. In addition, candidate functional genes potentially associated with biocontrol traits were investigated. This study provides a new fungal resource for the biological control of peanut RKN disease and a genomic foundation for further investigation of the molecular mechanisms underlying the biocontrol potential of *T. harzianum*.

## 2. Materials and Methods

### 2.1. Isolation and Culture Conditions of Fungal Strain

Fungi used here were isolated from peanut rhizosphere soil in Northeast China via the dilution plate method [29]. Sterile water was added to soil samples for homogenization to obtain suspensions. A serial dilution series was generated, and the resulting suspensions were spread onto potato dextrose agar (PDA) plates. After 5 days of incubation at 28 °C, individual fungal colonies were isolated and purified. Following incubation, the fungal culture broth was deposited at the Liaoning Provincial Germplasm Resource Bank of Plant Protection Microorganisms.

### 2.2. Morphological Observation and Molecular Identification of Strain Snef3255

Morphological observation was conducted to identify strain Snef3255. After cultivating single spores on PDA medium at 25 °C for 5 days, these plates were cultured in darkness at 25 °C for another 7 days. Colonial features and fungal microscopic structures were observed [30].

After 7 days of incubation on PDA medium, the mycelia of strain Snef3255 were collected and homogenized in liquid nitrogen. A 100 mg portion of the ground sample was loaded into a 2 mL centrifuge microtube. The HiPure Microbial DNA Kit (HiPure, Mumbai, India) was used to extract total genomic DNA from the strain following standard protocols. Molecular identification was performed by amplifying the ITS and *RPB2* gene fragments with specific primers (Supplementary Table S1) [31,32]. Detailed thermal profiles for PCR amplification are provided in Supplementary Table S2.

### 2.3. Inoculum Preparation of Meloidogyne hapla

*Meloidogyne hapla* (*M. hapla*) were collected from nematode-infested peanut fields at an experimental base (121°47′ E, 41°36′ N) in Beizhen, Liaoning Province, and reared on peanut seedlings in the greenhouse of the Northern China Nematology Institute, Shenyang Agricultural University. Egg masses were isolated from peanut roots following the protocol published by Martinuz et al. [33]. Eggs were hatched in sterile water at 25 °C for 5 days to obtain J2s, which were collected every 24 h. After dilution, the J2 suspension was standardized to 1000 J2/mL.

### 2.4. In Vitro Nematicidal Bioassay and Peanut Colonization Ability of T. harzianum Snef3255 Against M. hapla

Nematicidal activities of fungal isolates were detected using the J2s of *M. hapla*. Fermentation broth of the fungal isolate was obtained by culturing in PDB at an agitation speed of 150 rpm and 28 °C for 7 days, with a final spore concentration of 2 × 10^6^ spores/mL. After centrifugation at 4500× *g* for 15 min, the cell-free supernatant was collected and filtered using the filter (φ = 0.45 μm). To each plate, 200 μL of cell-free fungal supernatant at a series of concentrations (100%, 75%, 50%, 25%, and 10%) was added, along with 200 μL of J2 suspension. As the control group, only PDB medium was blended with the J2 suspension. Five replicates were established for every treatment, with all cultures incubated at 25 °C for 48 h. Counts of live and deceased J2s were recorded under a stereomicroscope 48 h following treatment. Individual nematodes were classified as dead upon showing morphological malformation and no movement in response to probing with a thin needle [34].

The J2 mortality was calculated as follows:

(1) J2 mortality (%) = Nd/NT × 100%

where Nd denotes the number of dead J2s, NT means total number of J2s.

The corrected J2 mortality was calculated as follows:

(2) Corrected J2 mortality (%) = [(MT − MC)/(1 − MC)] × 100%

where MT represents J2 mortality in the treatment group, and MC represents J2 mortality in the control group.

Genomic DNA was isolated from surface-sterilized peanut root samples harvested at the maturity stage. qPCR with TEF1-specific primers was applied to quantify *T. harzianum* Snef3255 colonization in peanut roots. Molecular identification was performed by amplifying the TEF1 gene fragments with specific primers (Supplementary Table S1). Fungal colonization abundance was calculated for each treatment using the pre-established standard curve.

### 2.5. Preparation of Seed-Coating Formulation and Seed Treatment

The auxiliary ingredients of the seed-coating formulation were obtained from previous laboratory studies, including binder, thickener, malachite green, and dispersant. Xanthan gum was applied as the binder at 1.5% (*w*/*v*). Modified starch served as the thickener at 2.2% (*w*/*v*). Malachite green was added at 1.0% (*w*/*v*) for warning colouration, and calcium lignosulfonate was used as the dispersant at 3.5% (*w*/*v*).

The combined seed-coating formulation (TC) was prepared using the fermentation broths of *T. harzianum* Snef3255, *Pseudomonas fluorescens* Sneb1910, and prepared auxiliary components. Firstly, the fermentation broth of Snef3255 was obtained by culturing in PDB medium at an agitation speed of 150 rpm and 28 °C for 3 days. The fermentation broth of Sneb1910, a strain previously screened in our laboratory with significant nematicidal activity, was produced by cultivation in LB medium at 180 rpm and 32 °C for 24 h. Secondly, the two fermentation broths were fully mixed at a volume ratio of 1:1 (*v*/*v*), and the mixture was further incubated at 150 rpm and 28 °C for 7 days for co-culture. Finally, the obtained co-cultured fermentation broth was blended with the prepared auxiliary components at a ratio of 1:1 (*v*/*v*) to produce the final TC seed-coating formulation. For the TC treatment, the TC was applied at 4 g formulation per kg seed.

The single seed-coating formulation (TS) was prepared using the fermentation broth of *T. harzianum* Snef3255 and prepared auxiliary components. The fermentation broth of Snef3255 was obtained by culturing in PDB medium at an agitation speed of 150 rpm and 28 °C for 7 days. Then, the obtained fermentation broth was blended with the prepared auxiliary components at a ratio of 1:1 (*v*/*v*) to produce the final TS seed-coating formulation. For the TS treatment, the TS was applied at 4 g formulation per kg seed.

Chemical control 1 (CH1) used 41.7% fluopyram SC at 0.3 g formulation/kg seed, while chemical control 2 (CH2) adopted 100 g L^−1^ abamectin SC at 1 g formulation/kg seed. Non-coated seeds were used for the CK treatment.

### 2.6. Biocontrol Assays for Root-Knot Nematode in the Pot Experiment and Field Experiment

The pot experiments were conducted in a greenhouse to evaluate the biocontrol efficacy of *T. harzianum* Snef3255 against *M. hapla*. Five treatments, namely TC, TS, CH1, CH2 and CK, were included in this assay. Approximately 2000 *M. hapla* J2s were inoculated into each pot 7 days after peanut seed sowing. The experiment was performed with five biological replicates. Five months later, 30 plants were randomly selected from each treatment to assess nematode control efficacy and yield.

The field experiment was performed from May to September 2024 at a site in Beizhen City, Liaoning Province, China (121°47′ E, 41°36′ N), where the soil had a severe natural infestation of *M. hapla*. The soil was characterized by a moderate sand–clay ratio, relatively flat terrain and deep soil layers, with the soil organic matter content ranging from 1% to 2%. This experimental site was selected based on the preliminary investigation because it had undergone 24 consecutive years of monocropping and suffered from a severe infestation of root-knot nematodes, which was suitable for evaluating the disease control and yield-increasing effects of the biocontrol fungi.

The field experiment was carried out on 1 May 2024. The peanut cultivar used was Luohanguo 807, provided by local farmers. Specifically, the initial population density of *M. hapla* J2s in the field soil before the trial was 102 nematodes per 200 mL soil. A randomized block design was adopted for the field plot experiment, with five treatments: TC, TS, CH1, CH2 and CK. Each treatment consisted of six plots, and the entire trial was established with five replicates. Each experimental plot had 3 ridges of 6 m in length, with 0.55 m between ridges, resulting in a plot area of 18 m^2^. A total of 100 peanut seeds were sown per ridge, and protective rows were set up around the experimental plots. All other field management practices were performed by local farmers according to conventional agricultural measures.

At the peanut maturity stage on 1 October 2024, multiple parameters including root gall index, egg masses/g root, eggs/g root, females/g root, J2 density/200 mL soil, and single-plant yield were measured.

Efficacy evaluation for root-knot nematode disease: Female adults of the nematode induce the formation of root knots on the peanut root surface. The number of root knots on the root system of a single plant was investigated, and the disease rating criteria for *M. hapla* are detailed in Supplementary Table S3 [35].

Gall index (GI) was computed via the weighted grading equation below:

(3) GI = [∑(gs × s)/(T × smax)] × 100

where s stands for root gall severity grade, gs indicates the count of plants bearing grade-s root galls, T means total surveyed peanut individuals, and smax refers to the highest level in the gall rating system.

Control efficacy (CE) was computed by the equation below:

(4) CE (100%) = [(Gick − GIt)/GIck] × 100%

where GIck means GI of CK; GIt indicates GI of treatment.

Determination of yield and percent yield increase: After peanut maturation, each plot was harvested individually. Fresh pod weight of each plot was recorded, and the average plot yield was calculated.

Percent yield increase (PYI) was calculated using the following equation:

(5) PYI (100%) = [(Yt − Yck)/Yck] × 100%

where Yt is the peanut pod yield of each treatment, and Yck refers to the yield of CK.

### 2.7. Sampling and DNA Extraction

Fungal mycelia were cultured on cellophane-covered PDA at 25 °C for 3 days. Whole genomic DNA was isolated using the SDS extraction method [36]. The integrity and concentration of DNA samples were determined using Qubit 2.0 fluorometric analysis. Purified high-quality DNA was delivered to Novogene for the construction of Illumina short-read and Nanopore long-read sequencing libraries.

### 2.8. Illumina, ONT and Hi-C Sequencing

Commercial whole-genome sequencing was performed using Oxford Nanopore PromethION (Oxford Nanopore Technologies, Oxford, UK) [37] and Illumina NovaSeq instruments (Illumina, San Diego, CA, USA). Qualified genomic DNA was sheared using G-tubes, followed by DNA damage repair and end preparation with NEBNext reagent modules (New England Biolabs, Ipswich, MA, USA). Sequencing adapters were attached according to the manufacturer’s protocol of the ONT ligation sequencing kit (Oxford Nanopore Technologies, Oxford, UK), and purification of the constructed libraries was performed with NZY Mag NGS magnetic beads (NZYtech, Lisbon, Portugal). Nanopore sequencing yielded 6.1 Gb of long-read data corresponding to approximately 150× genome coverage, whereas paired-end Illumina sequencing generated around 3.3 Gb short-read data.

Hi-C libraries were generated using cross-linked chromatin isolated from fungal mycelia, with library construction performed by Biomarker Technologies Co., Ltd. (Beijing, China). In brief, fungal mycelia were collected and cross-linked with 36% formaldehyde solution, followed by quenching with glycine. After cell lysis and nuclei isolation, the chromatin was subjected to restriction endonuclease digestion. Then the DNA ends were labeled with biotin and ligated. The ligated DNA was purified and fragmented to a size range of 200–600 bp and enriched using streptavidin magnetic beads. The qualified Hi-C libraries were subjected to Illumina sequencing, which yielded 150 bp paired-end high-quality reads. Hi-C data processing and chromosome scaffolding were carried out using LACHESIS (commit 1cf0f08, https://github.com/shendurelab/LACHESIS/blob/master/gnu_modulefile/lachesis, accessed on 14 August 2026) with specified parameters, and the interaction maps were further checked and adjusted manually [38]. Additionally, Hi-C library construction and sequencing were conducted, generating 4.1 Gb sequencing data (100× genome coverage) to assist subsequent genome assembly and scaffolding.

### 2.9. RNA Extraction and Sequencing

Total RNA was isolated from fungal mycelia using TriXtract reagent (MBC Biotech, St. Louis, MO, USA). The Kapa Stranded mRNA HyperPrep Kit (Roche, Wilmington, MA, USA) was adopted for constructing strand-oriented mRNA libraries, which were sequenced on an Illumina instrument by Biomarker Technologies Co., Ltd. (Beijing, China). FastQC v0.11 (https://gitcode.com/gh_mirrors/fa/FastQC, accessed on 27 February 2023) was implemented to examine raw read quality. Filtered RNA-Seq reads were mapped to the *T. harzianum* reference genome via HISAT2 v2.2.1 [39], and alignment results were subsequently evaluated using SAMTools v1.12 [40].

### 2.10. De Novo Assembly of T. harzianum Snef3255

The de novo long-read assembler Canu v1.5 [41] was used to assemble filtered Oxford Nanopore Technologies (ONT) subreads for error correction and generate draft contigs, which were further assembled using wtdbg2 v2.2 [42]. The draft genome assembly was sequentially refined and polished using Racon with filtered ONT reads and Pilon v1.22 [43] with Illumina short reads to improve the accuracy of the genome sequences. Clean reads were aligned to the genome using BWA [44]. The completeness of the assembled genome was evaluated by BUSCO [45] based on the fungi_odb9 database. For chromosome-scale scaffolding, Hi-C datasets were processed to anchor and arrange contigs using LACHESIS [38].

### 2.11. Genome Annotation

Gene structural annotation was implemented by integrating three analytical strategies, namely de novo prediction, homology-based annotation, and transcriptome-evidenced prediction. De novo gene prediction was performed using multiple bioinformatics tools, including Genscan [46], Augustus v2.4 [47], GlimmerHMM v3.0.4 [48], GeneID v1.4 [49], and SNAP [50]. Homology-dependent gene structure prediction was accomplished with GeMoMa v1.3.1 [51]. For transcriptome-supported gene identification, high-quality clean reads were aligned to the assembled genome via HISAT2 v2.0.4 [52], and transcript assembly was subsequently performed using StringTie v1.2.3 [52]. Transcript sequences were further identified and predicted using TransDecoder v2.0 [53] and PASA v2.0.2 [54]. Finally, gene models generated from the three prediction methods were comprehensively merged with EVM v1.1.1 [55], and the integrated gene models were further polished and optimized via PASA v2.0.2 [54].

Functional characterization of predicted genes from Snef3255 was conducted by aligning the gene sequences against the KOG [56], KEGG [57], Swiss-Prot [58], TrEMBL [58], and Nr databases [59] using BLAST v2.2.29 [60]. Based on the Nr BLAST results, GO annotation [61] was conducted using Blast2GO [62]. Pfam annotation was performed using HMMER [63] against the Pfam database [64]. In addition, enrichment analyses were performed for KOG categories, KEGG metabolic pathways, and GO terms.

Functional annotation of the Snef3255 predicted protein sequences was conducted via BLAST searches against multiple public databases, including the Transporter Classification Database (TCDB) [65], Pathogen-Host Interaction database (PHI) [66,67], Cytochrome P450 Engineering Database (CYPED) [68]. Carbohydrate-active enzyme genes were further characterized by screening the Carbohydrate-Active enZYmes (CAZy) [69] database with HMMER [70]. Prediction and characterization of secondary metabolic biosynthetic gene clusters were conducted with antiSMASH v8.0.4 [71].

Genomic tRNA loci were annotated using tRNAscan-SE v1.3.1 [72]. Other non-coding RNA members, such as rRNAs and other miscellaneous ncRNAs (excluding rRNAs and tRNAs), were identified using Infernal v1.1 [73] based on the Rfam v12.0 [64] database. For pesudogene identification, predicted protein sequences were initially matched to Swiss-Prot database with GenBlastA v1.0.1 [74] to retrieve homologous genomic sequences. Subsequently, GeneWise [75] was utilized to screen for premature termination codons and frameshift mutations, thereby enabling the final identification of pesudogenes.

### 2.12. Comparative Genomic Analysis

To investigate the genomic differences between *Trichoderma harzianum* strain Snef3255 and its closely related *Trichoderma* species, a comparative protein clustering analysis was conducted using the OrthoVenn3 web server [76]. The genome sequences of Snef3255 were aligned against four publicly available *Trichoderma* genomes acquired from the NCBI database, including *T. harzianum* CBS 226.95, *T. asperellum* FT101, *T. atroviride* P1, and *T. virens* Gv29-8. All protein sequences applied in this analysis were derived from the annotated whole-genome datasets, and the clustering procedure was performed according to the thresholds of sequence similarity and orthologous relationships.

Orthologous gene families across multiple fungal species (including the outgroup Botrytis cinerea and four *Trichoderma* species: *T. harzianum* CBS 226.95, *T. asperellum* FT101, *T. atroviride* P1, and *T. virens* Gv29-8) were clustered using OrthoFinder2 v2.5.4 [77], with only the longest coding isoform of each gene retained to remove sequence redundancy in preprocessing. Pairwise protein sequence alignment of all analyzed species was performed via DIAMOND [78] to infer interspecific sequence similarity. Based on the alignment results, OrthoFinder2 classified orthogroups into single-copy and multi-copy types. All filtered orthologous groups were aligned with MUSCLE v3.8.31 for multiple sequence comparison [79], and all aligned sequences were merged into a PHYLIP-formatted supergene matrix. ML phylogenetic tree was then constructed with RAxML v8.2.12 [80]. Orthogroups with a single gene copy in each tested species were defined as single-copy orthologs, and multi-copy orthologs were derived from evolutionary gene duplication. To ensure phylogenetic accuracy, single-copy proteins longer than 100 amino acids were screened and retained for tree reconstruction. The divergence time of these species was calculated with r8s v1.81 [81] and the mcmctree module embedded in PAML v4.10.0 [82], with calibration time points derived from TimeTree [83] and the published literature. The expansion and contraction patterns of orthogroups were examined using CAFE5 [84] according to the orthogroup clustering results. Additionally, ANI analysis was performed between strain Snef3255 and the four aforementioned *Trichoderma* species, with *B. cinerea* serving as the outgroup. Pairwise ANI statistics were generated with JSpeciesWS platform [85] based on the BLAST+ 2.2.29 (ANIb).

Whole-genome synteny analysis and identification of chromosomal rearrangements were performed using the MCScanX [86] program embedded in Tbtools IITBtools-II v2.471 [87], and phylogenetic tree visualization was conducted with iTOL [88]. The consensus phylogenetic tree was constructed using 1000 bootstrap replicates.

### 2.13. Prediction of Candidate Secreted Proteins from T. harzianum Snef3255

Genome-wide screening of secreted proteins was performed using the *T. harzianum* Snef3255 genome, from which Cerato-platanin and LysM were selected for functional analysis. Signal peptide regions were predicted using the online server SignalP 6.0 [89]. Transmembrane domains were annotated via TMHMM 2.0 [90]. Protein secondary structure was predicted with the SOPMA platform [91]. Protein tertiary structure analysis was conducted using SWISS-MODEL [92] and Phyre2 [93]. Conserved domains of target proteins were identified using the NCBI Conserved Domain Database (CDD) [94].

### 2.14. Establishment and Screening of Yeast Two-Hybrid Library

Total RNA was isolated from peanut tissues using TriXtract reagent (MBC Biotech, St. Louis, USA). First-strand cDNA was reverse-transcribed by Quantiscript Reverse Transcriptase (QIAGEN, Hilden, Germany). Double-stranded cDNA was obtained via LD-PCR amplification with the Make Your Own “Mate & Plate” Library System (Takara, Mountain View, CA, USA), normalized by DSN enzyme, and cloned into three-frame pGADT7-SmaI-1/2/3 via Infusion recombination. The construct was electroporated into Escherichia coli DH10B to generate the primary cDNA library, which was validated by titer determination and PCR.

Y2H library screening was performed according to the Coolaber Yeast Library Screening Manual (Beijing, China). Briefly, the peanut yeast library was screened via co-transformation of bait plasmids and library plasmids. The full-length cDNA sequences of *Thcp1* and *ThlysM1* were inserted into the pGBKT7 vector to construct recombinant bait plasmids pGBKT7-*Thcp1* and pGBKT7-*ThlysM1*. Autoactivation and toxicity assays were carried out by co-transforming each bait plasmid with the empty pGADT7 vector into Y2HGold yeast cells. Parallel treatments were arranged for the positive control (pGBKT7-53 paired with pGADT7-T) and negative control (pGBKT7-Lam paired with pGADT7-T). The transformed yeast suspensions were plated separately on two types of selective media: SD/-Leu/-Trp and SD/-Leu/-Trp/-His media. No autoactivation activity was observed. Qualified bait strains were then used for library screening via transformation with peanut cDNA library plasmids. Positive transformants were selected on SD/-Trp/-Leu/-His/-Ade medium. All primer sequences used for this research can be found in Supplementary Table S1.

Colonies with robust growth after 3–5 days of incubation at 30 °C were preliminarily screened as positive candidates. These positive single colonies were used as templates for PCR verification employing universal vector primers. Amplicons with clear and specific electrophoretic bands were recovered and subjected to Sanger sequencing. Obtained sequences of proteins potentially interacting with candidate secreted proteins were further aligned and annotated via the NCBI database.

### 2.15. Co-IP Assays

Co-IP assays were carried out to validate the in planta protein–protein interactions between ThCP1 and AhPR1, as well as between ThLysM1 and AhSRC2. Plant total proteins were extracted from fresh tissues using the Plant Total Protein Isolation Kit (Vivantis, Nallagandla, India). Subsequently, equal quantities of protein lysates were subjected to immunoprecipitation using the Protein A/G Magnetic Co-IP/IP Kit (APExBIO, Pune, India) following the manufacturer’s recommended procedures. Finally, the eluted protein samples were subjected to WB to confirm the pairwise protein interactions. The primer sequences are listed in Table S1.

### 2.16. BiFC Assays

For BiFC analyses, 4-week-old N. benthamiana leaves were agro-infiltrated with bacterial strains harbouring constructs encoding ThCP1-nYFP/AhPR1-cYFP and ThLysM1-nYFP/AhSRC2-cYFP fusion proteins. Leaf tissues were subjected to fluorescence imaging using an Olympus FV1000 confocal microscope two days post-agroinfiltration. The primer sequences are listed in Table S1.

### 2.17. Data Availability

The de novo assembled whole-genome sequences of *T. harzianum* Snef3255, as well as its ITS and *RPB2* gene sequences generated in this study, have been deposited in the GenBase [95] in National Genomics Data Center [96]. The whole-genome assembly is available under the accession number GWHISUI00000000.1 (BioProject ID: PRJCA062073; BioSample ID: SAMC7475426). The chromosomal sequences from Chr1 to Chr7 are assigned the accession numbers ranging from GWHISUI00000001.1 to GWHISUI00000007.1. Additionally, the ITS and *RPB2* gene sequences were deposited with the accession numbers C_AA219805.1 and C_AA168267.1, respectively. Both datasets are publicly accessible at https://ngdc.cncb.ac.cn (accessed on 14 August 2026).

### 2.18. Statistical Analysis

Statistical computations of experimental data were performed via SPSS 26.0. One-way ANOVA was conducted for multi-group statistical analysis. Different lowercase letters denote significant differences as determined by Duncan’s multiple-range test (*p* < 0.05).

## 3. Results

### 3.1. Morphological and Biological Characteristics of the Strain Snef3255

**Figure 1:**
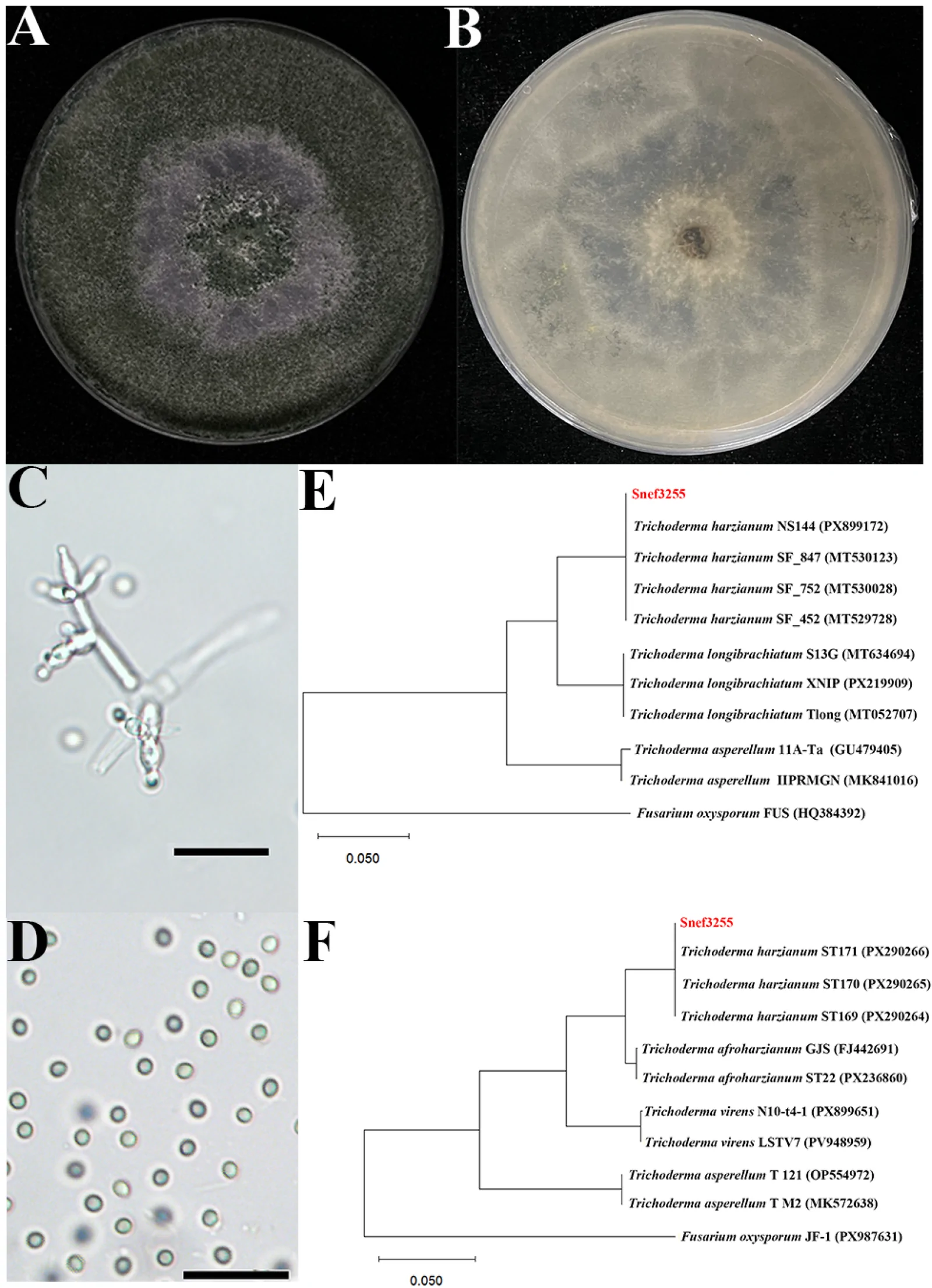
Colony morphology and conidiation of Snef3255. (**A**) Colony appearance of Snef3255 on PDA plate (upper view). (**B**) Colony appearance of Snef3255 on PDA plate (lower view). (**C**,**D**) Light micrographs of asexual reproductive structures, showing hyphae bearing conidiophores (**C**), conidiogenous (**D**). Scale bars = 20 μm (**C**,**D**). (**E**) Phylogenetic tree of strain Snef3255 using partial ITS nucleotide sequences. The scale bar represents 0.05 substitutions per nucleotide position. (**F**) Phylogenetic tree of strain Snef3255 using partial *RPB2* nucleotide sequences. The scale bar represents 0.05 substitutions per nucleotide position. The strain Snef3255 is marked in red.

*T. harzianum* strain Snef3255 was isolated from the rhizosphere soil of peanut grown under long-term continuous cropping in Liaoning Province, China. Morphological observation on the upper surface of the PDA plate showed that this strain initially formed colonies with sparse aerial hyphae and wide marginal bands, while concentric zones gradually appeared at the later incubation stage (Figure 1A). On the reverse side of PDA plates, colonies appeared yellowish-brown to dark brown, secreting soluble pigments that diffused into the surrounding agar matrix to generate a pale yellow halo (Figure 1B). Characteristically, the conidiophores formed a long axial structure that was fertile throughout to the tip, bearing solitary phialides on the main stem that were primarily intercalary and cylindrical, which occasionally became inflated in the middle and gathered in whorls atop the lateral branches (Figure 1C). The conidia exhibited an ellipsoidal morphology and a color spectrum spanning yellow–green to deep green, which could occasionally be accompanied by yellow pigmentation that developed in response to varying incubation environments (Figure 1D).

To clarify the taxonomic identity of strain Snef3255, we amplified and sequenced its ITS and *RPB2* gene regions. The ITS sequence was 565 bp and the *RPB2* sequence was 1071 bp. ITS and *RPB2* gene-based phylogenetic analysis using ML methods clearly identified Snef3255 as *T. harzianum* (Figure 1E,F).

### 3.2. Biocontrol Efficacy and Peanut Root Colonization Ability of T. harzianum Snef3255

**Figure 2:**
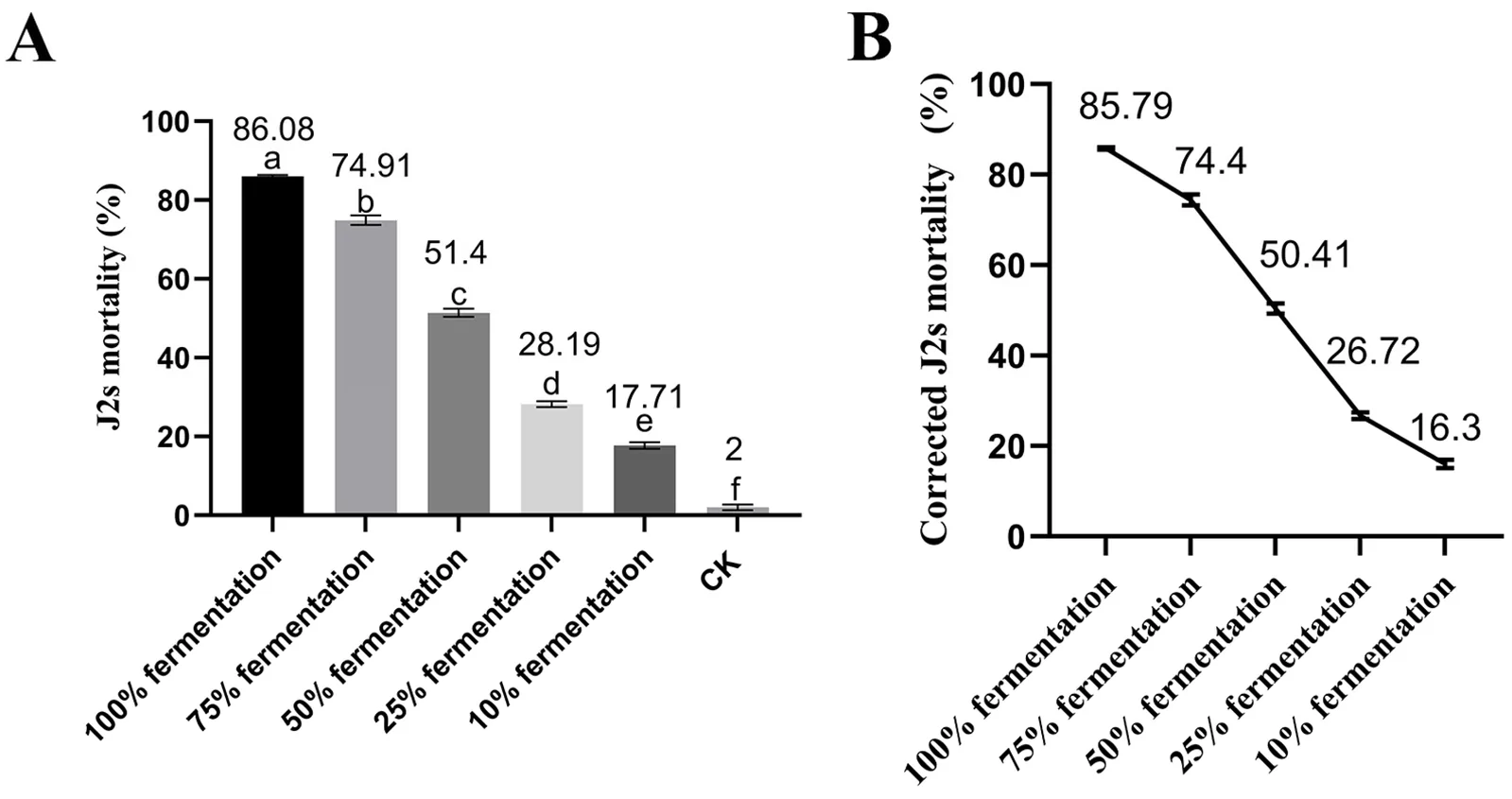
The effects of strain Snef3255 on J2 mortality and corrected J2 mortality of *M. hapla* in vitro. (**A**) The effects of strain Snef3255 on J2s mortality of *M. hapla* in vitro. (**B**) The effects of strain Snef3255 on corrected J2s mortality of *M. hapla* in vitro. Different lowercase letters denote significant differences as determined by Duncan’s multiple range test (*p* < 0.05).

At 48 h, the cell-free fermentation supernatant of *T. harzianum* strain Snef3255 at a concentration of 100% exhibited high virulence against J2s of *Meloidogyne hapla*, with a J2 mortality of 86.08%. The J2s mortalities at concentrations of 75%, 50%, 25%, 10% and for the CK group were 74.91%, 51.40%, 28.19%, 17.71%, and 2.00%, respectively (Figure 2A). The corresponding corrected J2s mortality values at supernatant concentrations of 75%, 50%, 25%, and 10% were 85.79%, 74.40%, 50.41%, and 16.30%, respectively (Figure 2B).

**Figure 3:**
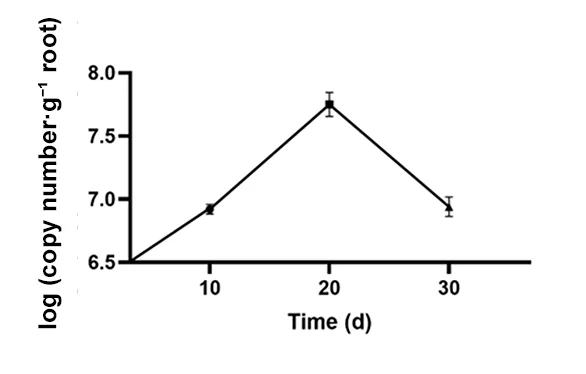
Quantification of *T. harzianum* Snef3255 in peanut roots with cDNA synthesized from isolated RNA (values are shown as the log_10_ conversion of the TEF1 copies g^−1^ root).

Over four sampling time points, cDNA-based quantitative real-time qPCR was performed to investigate the root colonization of *T. harzianum* Snef3255 in peanut roots. Detectable colonization of the fungus was observed in peanut roots at 10 days post-inoculation (dpi). As time elapsed, the root colonization abundance continuously increased and reached a peak value of 7.75 (log_10_ copy number g^−1^ root) at 20 dpi, followed by a marked decrease in fungal biomass inside the roots. Collectively, *T. harzianum* Snef3255 could successfully colonize peanut roots and achieved the maximum colonization abundance at 20 dpi. Under the combined effects of plant immunity and rhizosphere micro-environmental competition, the colonized fungal biomass subsequently decreased and remained at a relatively steady level (Figure 3).

### 3.3. Biocontrol Efficacy Against Root-Knot Nematoe in the Pot Experiment and Field Experiment

To explore the biocontrol potential of *T. harzianum* Snef3255 against *M. hapla*, peanut seeds were treated with seed coating agents TC and TS before inoculation with J2 of *M. hapla*. Five months after nematode inoculation, the root-knot indices of the TC and TS treatments were significantly lower than those of CK and CH2, with significant differences also observed between TC and TS, which were 20 and 24.67, respectively. The biocontrol efficacies of TC and TS were significantly higher than that of CH2 and differed significantly from each other, reaching 73.22% and 66.97%, respectively. Compared with the CK group, both TC and TS treatments exerted prominent suppressive effects on root-knot nematode reproduction and soil nematode populations. Consistently, TC and TS exhibited significantly lower values than the CK group in terms of the number of egg masses per gram of root, eggs per gram of root, populations of female nematodes, and J2 density per 200 mL soil. The peanut yields of TC and TS treatments were significantly higher than those of CK and CH2, which were 41.04 g/plant and 39.42 g/plant, respectively. The yield increase rates of TC and TS were significantly higher than that of CH2, being 18.61% and 13.92%, respectively. Although CH1 exhibited a slightly higher biocontrol efficacy than TC, no significant difference in yield increase rate was found between CH1 and TC (Table 1).

The field experiments showed similar results to the greenhouse pot experiments. To explore the biocontrol potential of *T. harzianum* Snef3255 against *M. hapla*, peanut seeds were treated with seed coating agents TC and TS before inoculation with J2 of *M. hapla*. Five months after nematode inoculation, the root-knot indices of the TC and TS treatments were significantly lower than those of CK and CH2, with significant differences also observed between TC and TS, which were 21.67 and 28.33, respectively. The biocontrol efficacies of TC and TS were significantly higher than that of CH2 and differed significantly from each other, reaching 70.85% and 61.88%, respectively. Compared with the CK group, both TC and TS treatments exerted prominent suppressive effects on root-knot nematode reproduction and soil nematode populations. Consistently, TC and TS exhibited significantly lower values than the CK group in terms of the number of egg masses per gram of root, eggs per gram of root, populations of female nematodes, and J2 density per 200 mL soil. The peanut yields of TC and TS treatments were significantly higher than those of CK and CH2, which were 40.73 g/plant and 39.16 g/plant, respectively. The yield increase rates of TC and TS were significantly higher than that of CH2, being 18.15% and 13.61%, respectively. Although CH1 exhibited a slightly higher biocontrol efficacy than TC, no significant difference in yield increase rate was found between CH1 and TC (Table 2).

### 3.4. Genome Sequencing, Assembly and Structural Annotation

A total of 6.1 Gb ONT long-read sequencing data (~150× genome coverage) and ~3.3 Gb Illumina short-read sequencing data were obtained; 4.1 Gb sequencing data (100× genome coverage) were generated by Hi-C library. Long reads were de novo assembled and corrected, the short reads were used for polishing, and Hi-C data was used to anchor and refine contigs. The final assembly comprised seven contigs, with an N50 of ~6.3 Mb (Table 3). Short-read alignment showed that 99% of the reads mapped to the assembled genome. Furthermore, BUSCO analysis indicated a completeness score of 99.31%.

**Figure 4:**
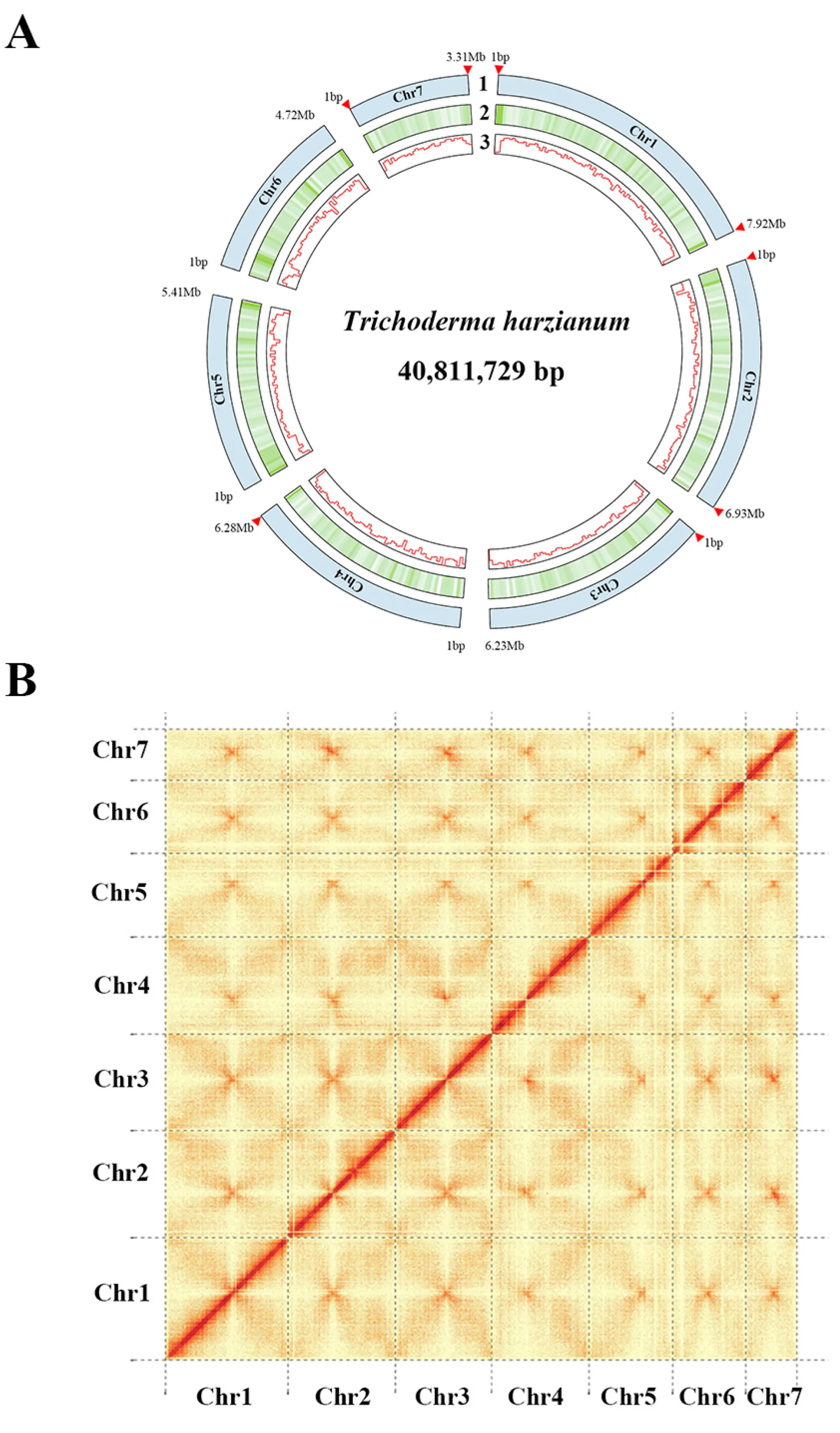
Genomic characteristics of *T. harzianum* Snef3255. (**A**) Circular genome map of *T. harzianum* Snef3255. Track 1 represents the seven assembled chromosomes, with their respective sizes labeled. The red arrow markers at chromosomal termini represent telomeric repetitive sequences. Tracks 2 and 3 separately show the chromosomal distribution of gene density and GC density. (**B**) An Illustration of genome-wide chromatin interaction patterns characterized by Hi-C.

The seven chromosomes were chromosome-level assemblies, with a total length of ~40.8 Mb (Table 3, Figure 4). The longest chromosome was 7.9 Mb, and the shortest was 3.3 Mb. The telomere repeat sequences were detected at both ends of chromosome 1, 2, and 7, whereas only the 5′ end was detected on chromosome 3 and only the 3′ end was detected on chromosome 4. These findings reveal the high quality and continuity of the genome assembly.

### 3.5. Genome Annotation and Genomic Features

The assembled genome was annotated by PASA and EVM pipeline. A total of 13,611 protein-coding genes were predicted, with an average length of ~1.95 kb (Table 4). In addition, 39,296 exons were identified, with an average of 2.89 exons per gene. The genome also contained 332 non-coding genes, including 223 tRNA genes, 74 rRNA genes and 35 other non-coding genes.

**Figure 5:**
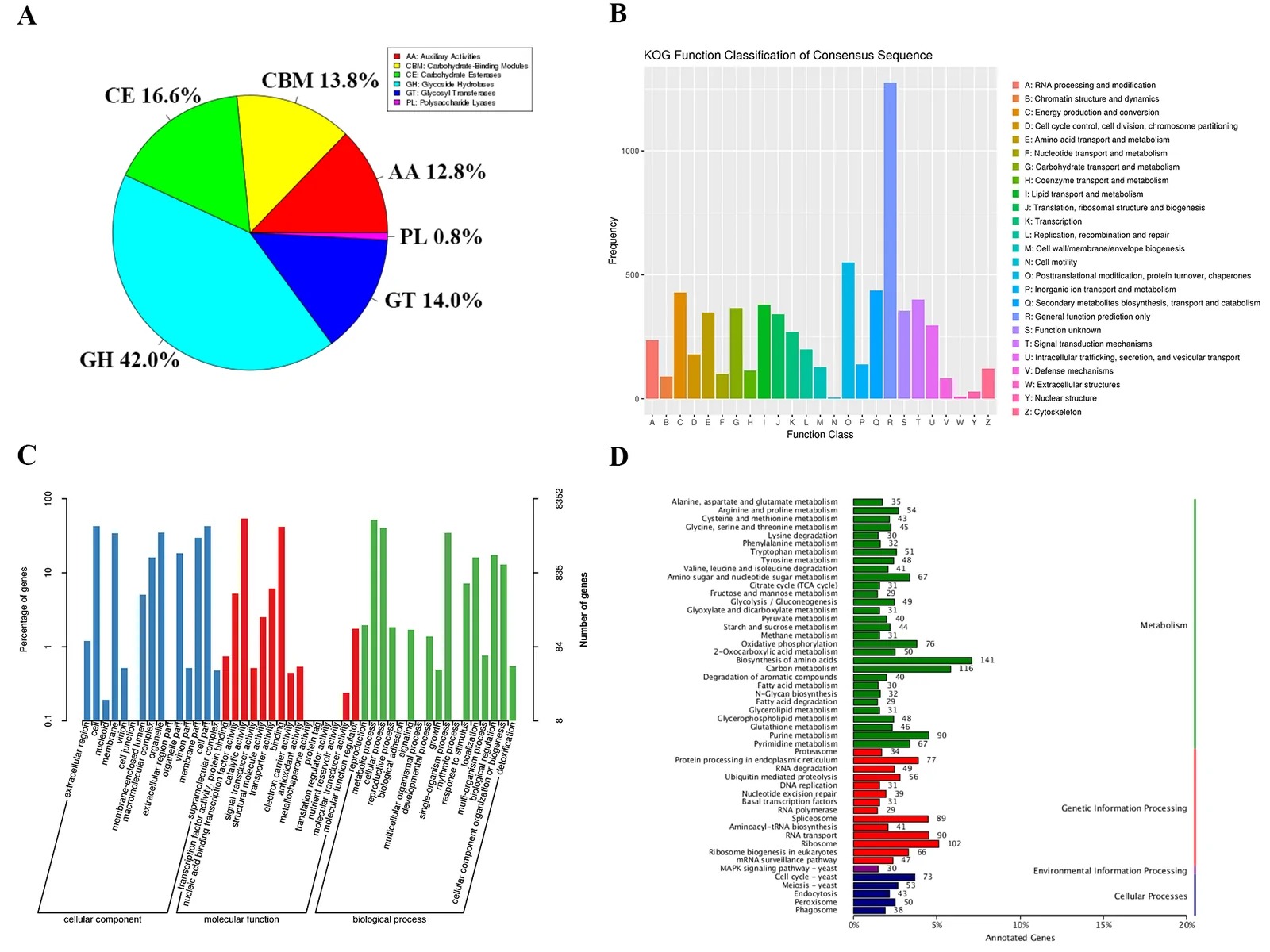
The functional annotation and gene prediction results for the strain Snef3255. (**A**) CAZy functional classification. (**B**) KOG functional classification. (**C**,**D**) Gene distribution based on GO and KEGG pathways.

Functional annotation of all protein-coding genes was completed across multiple databases, including nr, TrEMBL, Swiss-Prot, Pfam, and so on. Overall, 12,096 genes were annotated in at least one database (Table 4). Among them, 627 genes were annotated as CAZymes, of which glycoside hydrolases (GH) constituted the largest class (Figure 5A). Moreover, 8352 and 3379 genes were assigned GO terms and KEGG pathways, respectively (Figure 5B–D). The enrichment of genes involved in cell wall degradation, metabolism, and environmental adaptation provided putative genomic support for the potential of this strain as a highly efficient biocontrol agent against RKNs (Figure 5). In addition, 521 genes were annotated as members of cytochrome P450 families. Furthermore, a total of 3929 genes showed homology to entries in the PHI database, and a total of 53 secondary metabolite biosynthesis gene clusters were predicted via antiSMASH, indicating that these genes and clusters may contribute to fungal virulence.

### 3.6. Comparative Genomic and Phylogenetic Analyses

**Figure 6:**
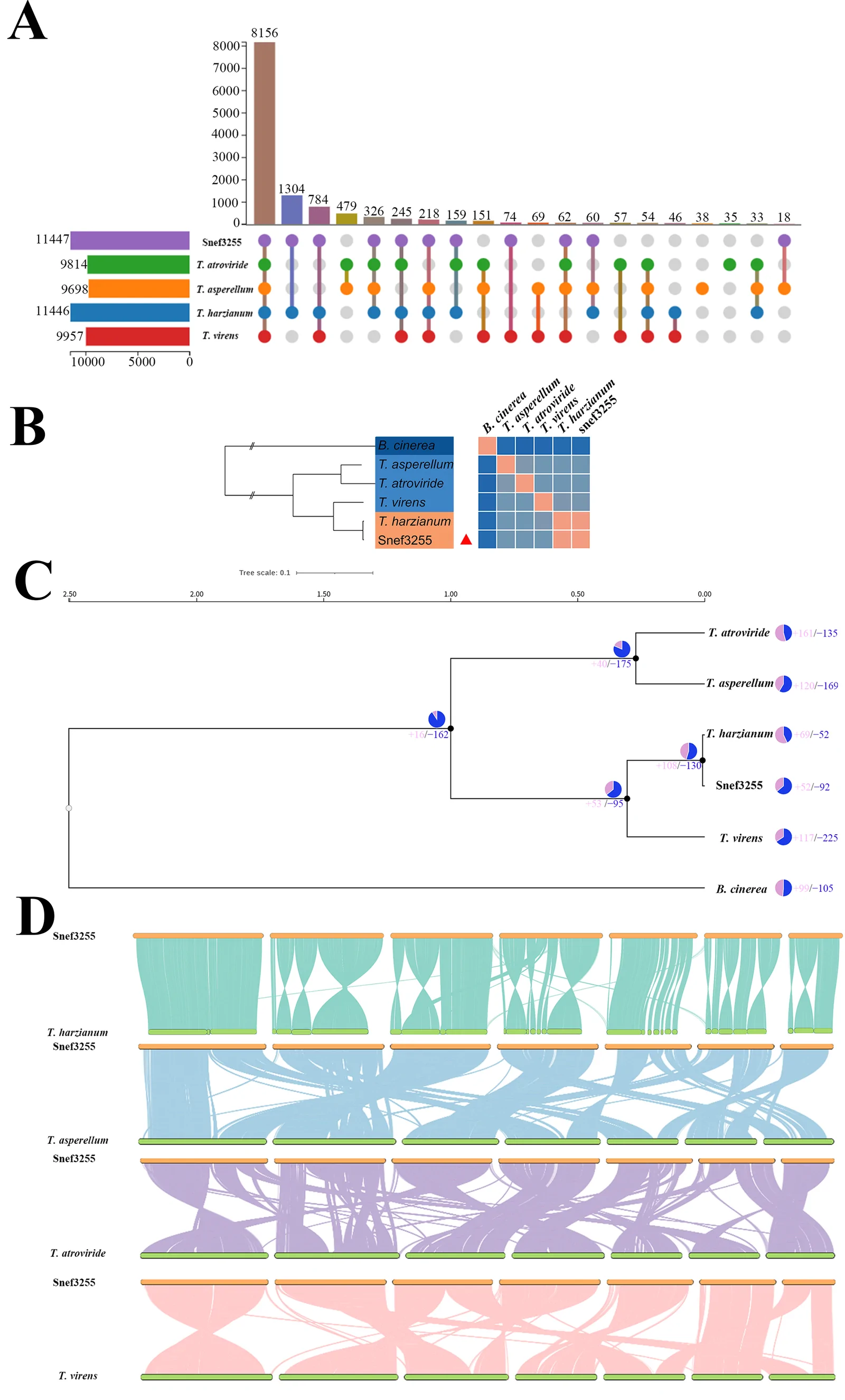
Comparative genomic and phylogenetic characteristics of *T. harzianum* Snef3255 and its closely related fungal species. (**A**) Gene family clustering of *T. harzianum* Snef3255 and other *Trichoderma* species. (**B**) ML phylogenetic tree constructed from single-copy orthologs. The heatmap shows pairwise ANI values among all strains. (**C**) Divergence time estimation of *Trichoderma* species. The median divergence time between Snef3255 and the reference *T. harzianum* strain was estimated to be ~0.02 Ma. The corresponding 95% highest-posterior-density (HPD) confidence interval was 0.007–0.041 Ma. (**D**) Genome synteny analysis between *T. harzianum* Snef3255 and three closely related species.

To characterize shared and strain-specific genomic features, we compared the genome of *T. harzianum* strain Snef3255 with those of four publicly available *Trichoderma* strains via the OrthoVenn3 platform. This analysis identified a core set of 8156 orthologous gene clusters, that were conserved across all five strains included in this study (Figure 6A).

Notably, functional annotation revealed two strain-specific genes in *Trichoderma* Snef3255 that confer unique metabolic and adaptive characteristics. Protein Q4WTT9, encoded by Region 6.3, belongs to the cytochrome P450 superfamily and participates in xenobiotic metabolism. This protein endows the strain with capacities for detoxification, exogenous compound degradation, and competitive colonization within soil environments. Region 2.7 encodes protein A2EYC4, which is located within an uncharacterized gene cluster. Collectively, these two unique gene clusters may endow Snef3255 with superior environmental adaptability and distinctive biocontrol capacity relative to other Trichoderma strains (Table 5). Furthermore, conserved secondary metabolic gene clusters, including the NRPS biosynthetic gene cluster and the 6-PP-synthesizing PKS1 biosynthetic gene cluster, produce diverse bioactive secondary metabolites. These metabolites are speculated to possess nematicidal activities and xenobiotic detoxification functions. More importantly, these conserved gene families may collaboratively shape the nematode-suppressive capacity of *Trichoderma*, providing a potential core genetic basis for its growth adaptation, biocontrol performance against plant-parasitic nematodes, and multitrophic interaction with hosts and nematodes (Table 6).

Phylogenetic tree construction and molecular dating analysis were conducted using single-copy orthologous genes conserved across *Trichoderma* species. Phylogenetic analysis confirmed that all *Trichoderma* species formed a monophyletic clade, with Snef3255 nested within the *T. harzianum* lineage. Notably, Snef3255 showed the closest phylogenetic relationship with *T. harzianum*, with an ANI value as high as 99.6% (Figure 6B), diverging from their most recent common ancestor approximately 0.02 Ma ago (Figure 6C).

Whole-genome collinearity analysis showed that *T. harzianum* Snef3255 exhibited more extensive conserved synteny with the *T. harzianum* strain than with the other three *Trichoderma* species. Nevertheless, certain structural rearrangements (translocations, inversions, and deletions) were still detected among these *Trichoderma* species (Figure 6D). These findings not only confirm the taxonomic status and evolutionary affinity of *T. harzianum* Snef3255 at the genomic level, but also lay a key foundation for exploring genes related to its biocontrol-associated biological characteristics.

### 3.7. Identification and Cloning of Candidate Secreted Proteins from T. harzianum Snef3255

**Figure 7:**
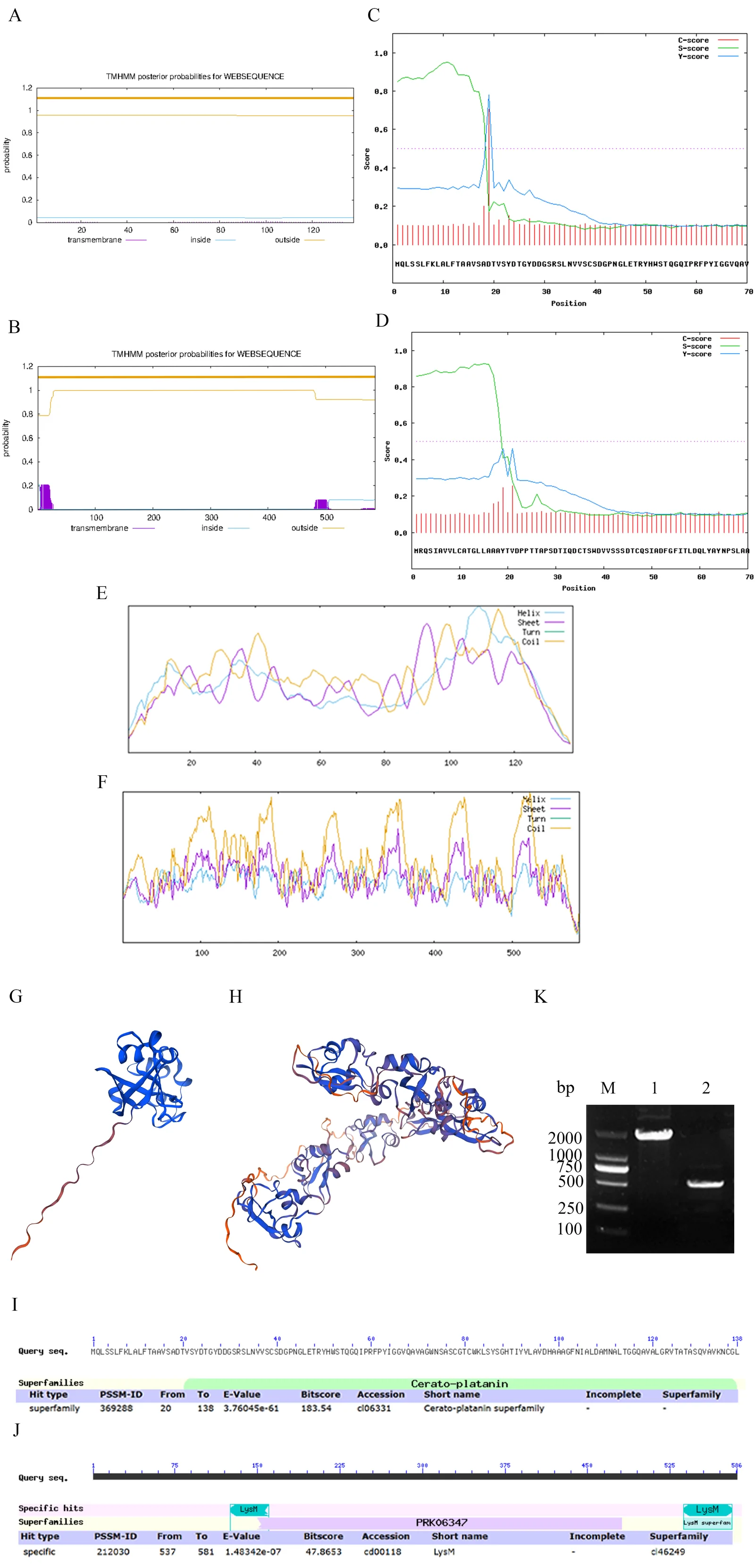
Identification and cloning of *Thcp1* and *ThlysM1* genes and bioinformatic prediction of their encoded proteins. (**A**,**B**) Transmembrane domain prediction of ThCP1 and ThLysM1 using TMHMM 2.0, respectively. (**C**,**D**) Signal peptide prediction of ThCP1 and ThLysM1 using SignalP-6.0, respectively. (**E**,**F**) Secondary structure prediction of ThCP1 and ThLysM1 using SOPMA, respectively. (**G**,**H**) Three-dimensional structural model of ThCP1 and ThLysM1 constructed by SWISS-MODEL, respectively. (**I**,**J**) Conserved domain analysis of ThCP1 and ThLysM1 using the NCBI Conserved Domain Database (CDD), respectively. (**K**) Agarose gel electrophoresis of the cloned ThCP1 and ThLysM1 genes. M: DNA Marker DL2000; *ThlysM1*; 2: *Thcp1*.

As the CP1 and LysM1 have been previously identified as key functional secreted proteins in *T. atroviride* and *T. virens* [26,27], their homologs in *T. harzianum* Snef3255, ThCP1 and ThLysM1, were selected as putative secreted proteins for further characterization. Transmembrane domain prediction showed that neither ThCP1 nor ThLysM1 contained transmembrane helices, and both proteins were predicted to be extracellularly localized, consistent with the characteristics of secreted proteins (Figure 7A,B).

Signal peptide prediction further indicated that both proteins possess N-terminal signal peptides. The predicted cleavage site of ThCP1 was located between residues 17 and 18, with the signal peptide sequence MQLSSLFKLALFTAAVS (Figure 7C), whereas that of ThLysM1 was located between residues 18 and 19, with the sequence MRQSIAVVLCATGLLAAA (Figure 7D). Secondary structure analysis indicated that both proteins were dominated by random coils, together with alpha helices and extended strands (Figure 7E,F). Tertiary structure modeling revealed high sequence identity between ThCP1 and the *T. virens* Epl1 template (91.67%), and between ThLysM1 and a LysM domain-containing protein from *T. guizhouense* (97.61%), supporting the high reliability of the predicted structures (Figure 7G,H). Conserved domain analysis further showed that ThCP1 contains a Cerato-platanin family domain, whereas ThLysM1 contains a LysM domain (Figure 7I,J). Furthermore, the two genes were successfully cloned, with lengths of 480 bp and 1923 bp, respectively (Figure 7K).

### 3.8. Construction of a Yeast Two-Hybrid cDNA Library for Peanut and Screening for Proteins Interacting with Candidate Secreted Proteins ThCP1 and ThLysM1

**Figure 8:**
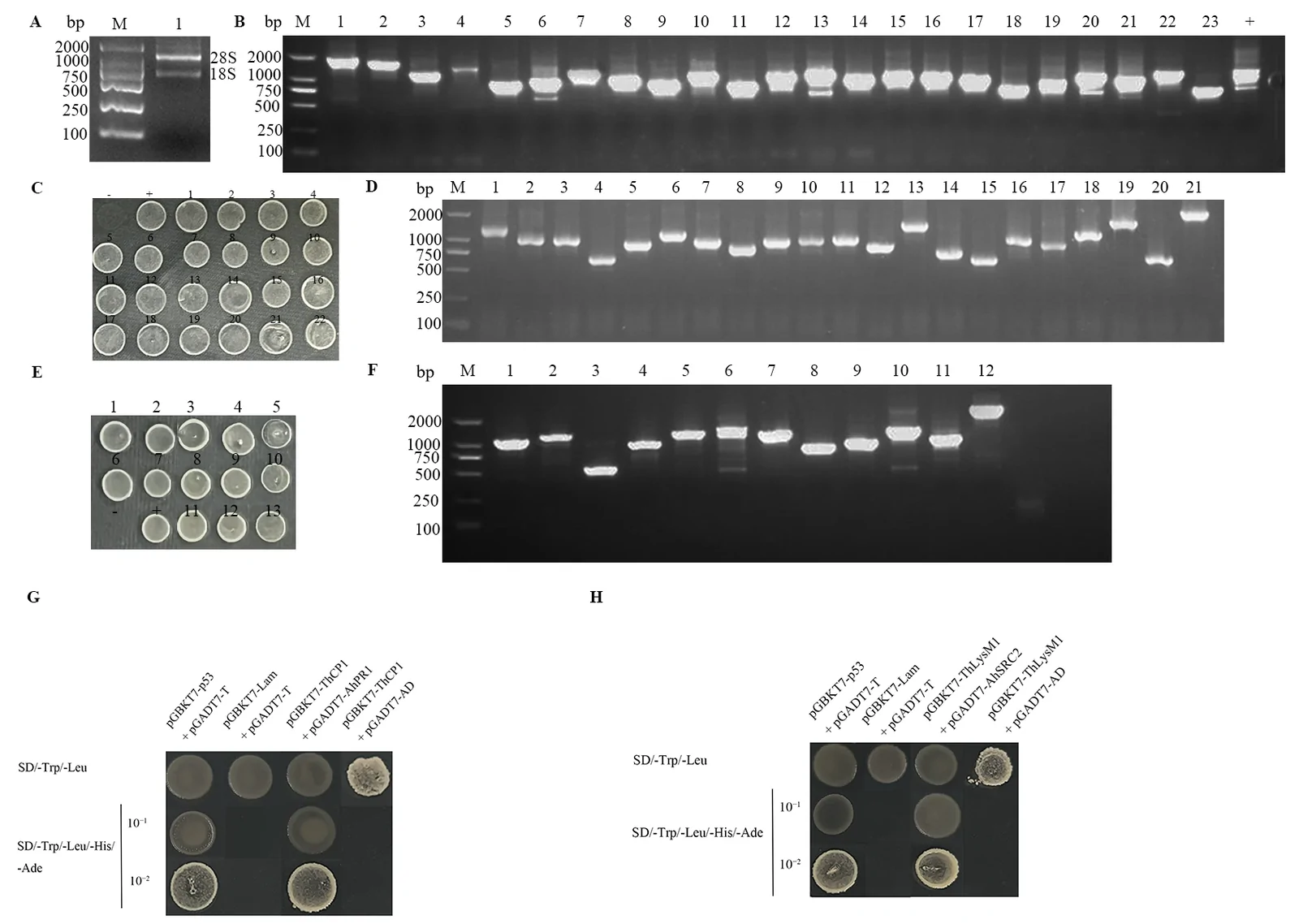
Construction of a yeast two-hybrid cDNA library for peanut and screening for proteins interacting with candidate secreted proteins of *T. harzianum* Snef3255. (**A**) Agarose gel electrophoresis of total RNA extracted from peanut. M: DNA Marker DL2000; 1: total RNA. (**B**) PCR identification of inserted fragments from 23 clones in the library. M: DNA Marker DL2000; 1–23:23 clones. (**C**) Spot plate screening of positive clones for ThCP1-interacting proteins on SD/-Trp/-Leu/-His/-Ade plates. (**D**) PCR verification of prey plasmids from positive yeast clones for ThCP1-interacting proteins. M: DNA Marker DL2000; 1–21:21 clones. (**E**) Spot plate screening of positive clones for ThLysM1-interacting proteins on SD/-Trp/-Leu/-His/-Ade plates. (**F**) PCR verification of prey plasmids from positive yeast clones for ThLysM1-interacting proteins. M: DNA Marker DL2000; 1–12:12 clones. (**G**) Pairwise validation of ThCP1 candidate interacting proteins. (**H**) Pairwise validation of ThLysM1 candidate interacting proteins.

Total RNA extracted from peanut roots exhibited distinct 28S and 18S ribosomal RNA bands that featured the 28S band with significantly greater intensity than the 18S band, which verifies high RNA integrity with a RIN value exceeding 8.0 (Figure 8A). The library titer reached 2  ×  10^7^ CFU·mL^−1^. Colony PCR analysis of 23 randomly selected clones from the library showed that the recombination efficiency reached 100% with an average insert size of over 1000 bp (Figure 8B), which satisfied the standard quality criteria for Y2H screening.

To identify proteins interacting with the secreted protein CP1 and LysM1 from *T. harzianum* Snef3255, peanut yeast cDNA library plasmids were co-transformed with pGBKT7-*Thcp1* or pGBKT7-*ThlysM1* bait plasmids into yeast competent cells, respectively. Transformation efficiency was determined by plating 100 μL of 10-fold diluted transformed cultures on SD/-Trp/-Leu plates. Colonies with potential interactions were further screened on SD/-Trp/-Leu/-His/-Ade plates (Figure 8C,E); colored colonies were subjected to colony PCR, and PCR products with distinct bands were sequenced (Figure 8D,F). BLAST analysis against the NCBI database identified 21 putative ThCP1-interacting proteins and 12 putative ThLysM1-interacting proteins from sequencing results.

To validate protein interactions with ThCP1 and ThLysM1, the full-length CDS of two candidates for each target were cloned into pGADT7. All yeast transformants grew on SD/-Trp/-Leu plates. On SD/-Trp/-Leu/-His/-Ade plates, the negative control showed no colony growth, while the positive control and ThCP1-interacting candidates (AhPR1) grew robustly (Figure 8G), and ThLysM1-interacting candidates (AhSRC2) exhibited vigorous growth (Figure 8H), confirming direct interactions of these candidates with ThCP1 and ThLysM1, respectively.

**Figure 9:**
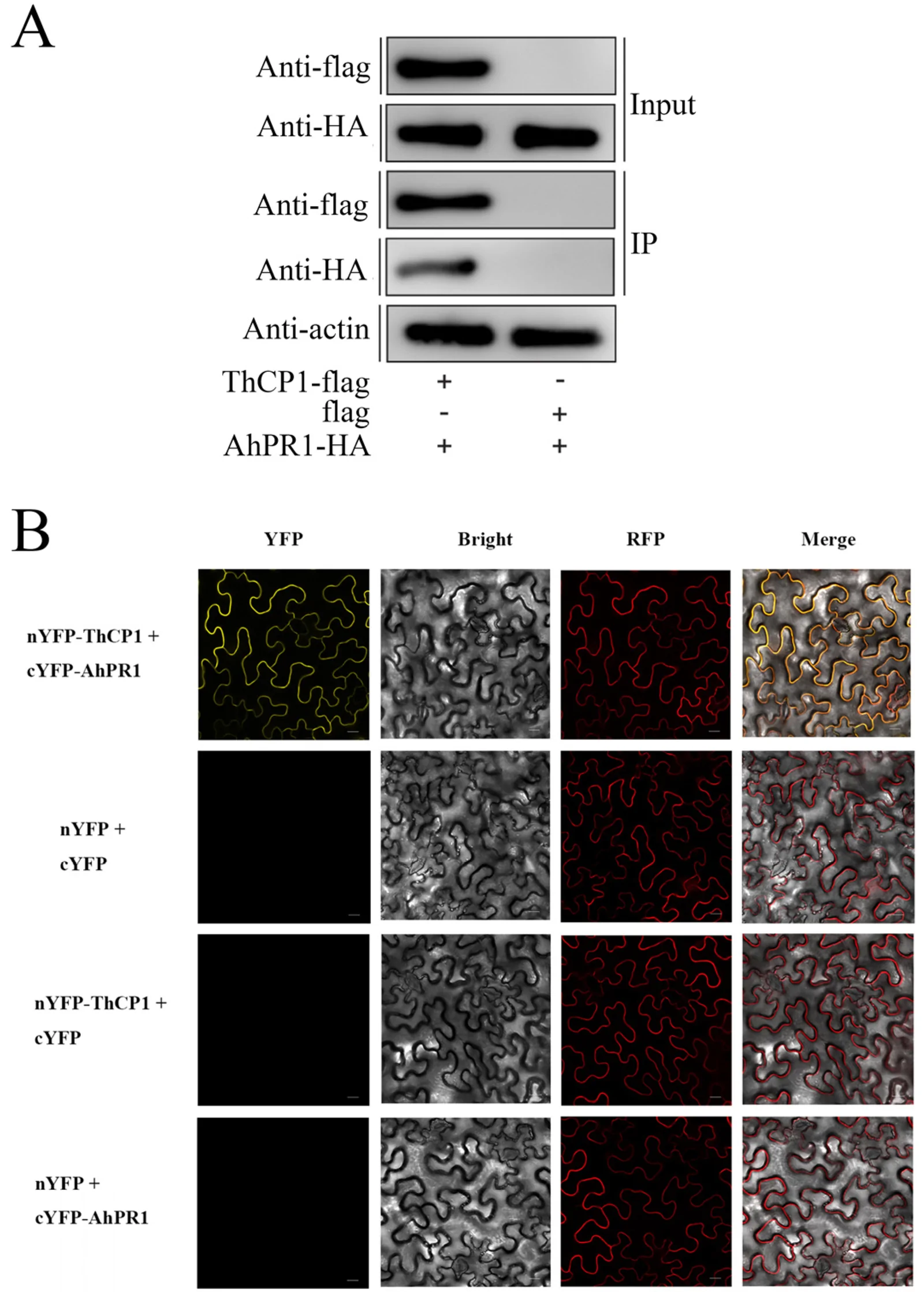
Interaction of ThCP1 and AhPR1. (**A**) Co-IP confirmed that ThCP1 was associated with AhPR1 in plant cell. (**B**) BiFC confirmed the interaction of ThCP1 and AhPR1 in vivo. YFP channel shows yellow fluorescence signal derived from reconstituted YFP; RFP channel displays red fluorescence from the plasma-membrane marker; Bright represents bright-field images showing cell morphology; Merge indicates the merged image of YFP, RFP and bright-field channels. nYFP-ThCP1 + cYFP-AhPR1 represents the experimental group; nYFP + cYFP, nYFP-ThCP1 + cYFP and nYFP + cYFP-AhPR1 are negative control groups. Scale bars = 20 μm.

Co-IP experiments validated the physical interaction between ThCP1 and AhPR1 within plant cells (Figure 9A). This protein–protein association was further supported by BiFC assays performed in planta. The fusion constructs nYFP-ThCP1 and cYFP-AhPR1 were transiently co-expressed in *N. benthamiana* leaf tissues. Distinct yellow YFP fluorescence was visualized at the cell membrane in Agrobacterium-infiltrated leaf cells. Conversely, none of the negative-control combinations produced detectable fluorescence signals, even though all fusion proteins accumulated to adequate levels in planta. These results demonstrated that ThCP1 and AhPR1 physically interact at the plant cell membrane (Figure 9B).

**Figure 10:**
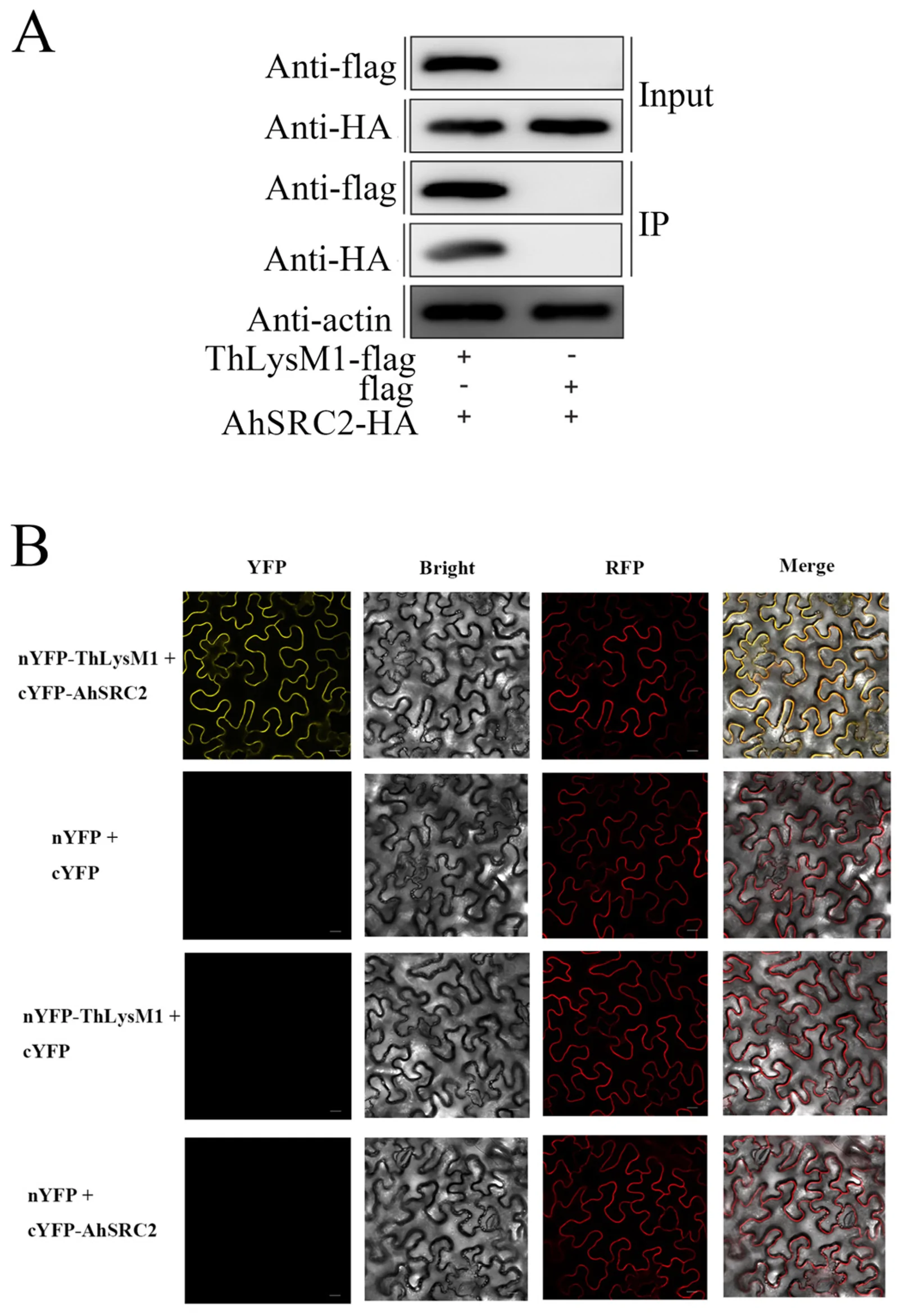
Interaction of ThLysM1 and AhSRC2. (**A**) Co-IP confirmed that ThLysM1 was associated with AhSRC2 in planta. (**B**) BiFC confirmed the interaction of ThLysM1 and AhSRC2 in vivo. YFP channel shows yellow fluorescence signal derived from reconstituted YFP; RFP channel displays red fluorescence from the plasma-membrane marker; Bright represents bright-field images showing cell morphology; Merge indicates the merged image of YFP, RFP and bright-field channels. nYFP-ThCP1 + cYFP-AhPR1 represents the experimental group; nYFP + cYFP, nYFP-ThCP1 + cYFP and nYFP + cYFP-AhPR1 are negative control groups. Scale bars = 20 μm.

Likewise, Co-IP experiments verified the physical interaction between ThLysM1 and AhSRC2 inside plant cells (Figure 10A). BiFC assays carried out in planta further corroborated this protein–protein interaction. The fusion constructs nYFP-ThLysM1 and cYFP-AhSRC2 were transiently co-expressed in *N. benthamiana* leaf tissues. Clear yellow YFP fluorescence could be observed at the cell membrane of Agrobacterium-infiltrated leaf cells. In contrast, no detectable fluorescence was generated for any of the negative-control combinations, despite sufficient accumulation of all fusion proteins in planta. Collectively, these findings revealed that ThLysM1 physically interacts with AhSRC2 at the plant cell membrane (Figure 10B).

## 4. Discussion

Peanut is a globally important oilseed crop whose yield stability is severely threatened by RKNs [1,2]. Previous reports have shown that *Trichoderma* antagonizes *M. arenaria*, *M. incognita* and *M. javanica* [3,4,5,97]. However, related assessments on its biocontrol efficacy against *M. hapla* are still insufficient [6]. There is thus an urgent demand for eco-friendly alternatives, such as microbial biocontrol agents and induced host resistance [7]. In this study, the *T. harzianum* strain Snef3255 was isolated from peanut rhizosphere soil and showed prominent nematicidal activity against *M. hapla*. This strain also exhibited strong field control efficacy against peanut RKN and significantly increased peanut yield. Meanwhile, we constructed a high-quality chromosome-level genome of Snef3255, performed comparative genomic analysis, and identified two key secreted proteins, ThCP1 and ThLysM1, which provided a genomic foundation for exploring the interaction mechanism between *T. harzianum* and peanut.

Many studies have confirmed that different *Trichoderma* strains exhibit considerable variation in biocontrol efficacy against RKNs, and the control effect varies greatly with application methods and target nematode species [10,98,99,100,101]. For instance, *Trichoderma* formulations inhibited the egg hatching of *M. arenaria* by 62.1–83.7% in vitro [9]. *T. harzianum* exerted strong inhibition on egg hatching of *M. incognita*, reaching an inhibition rate of 86.50% after 7 d of incubation [4]. Under field conditions, peanut treated with integrated microorganisms including *T. harzianum* exhibited a 62.7% reduction in root galling induced by *M. javanica* [8]. In this study, we isolated the indigenous *T. harzianum* strain Snef3255. Field trials revealed that its TSC and TFB formulations exhibited control efficacies of 70.85% and 61.88% against *M. hapla*, with peanut yields increased by 18.15% and 13.61%, respectively. To our knowledge, this is the first report of describing nematicidal activity of *T. harzianum* against *M. hapla* in peanut. Notably, the field trials in this study were conducted within a single growing season. Because the field performance of microbial biocontrol agents can vary with climatic and soil conditions, the present results should be considered preliminary. Multi-season field trials are therefore needed to further evaluate the stability and practical applicability of Snef3255 formulations.

In recent years, whole-genome sequencing has been widely used to dissect the biocontrol mechanisms of *Trichoderma*, and high-quality genome assembly is the foundation for revealing the genetic basis of biocontrol traits [14,99,102,103,104,105]. Most of the available *T. harzianum* genomes are the scaffold-level assemblies, and high-completeness chromosome-level genomes are still scarce, limiting in-depth analyses of gene structure, comparative genomics, and functional gene mining [15,16,17,18,19,20]. In this study, we generated a chromosome-level genome assembly of *T. harzianum* strain Snef3255 by integrating ONT, Illumina, and Hi-C sequencing technologies. The resulting assembly exhibited high continuity, completeness, and accuracy, representing one of the high-quality *T. harzianum* genomes reported to date. This genomic resource provides an important foundation for systematically investigating the biocontrol mechanisms of Snef3255 against *M. hapla*.

Comparative genomic studies have found that there are a large number of conserved core gene families among different *Trichoderma* species, while species-specific or strain-specific genes are often related to niche adaptation and unique biocontrol characteristics [21,22,106]. Comparative genomic analysis in this study confirmed that Snef3255 belonged to the *T. harzianum* evolutionary clade and showed high collinearity with the reference strain, but also harbored specific structural variations and unique gene clusters, which may be related to its strong nematicidal activity and adaptability to the peanut rhizosphere. These results help to understand the evolutionary basis of the biocontrol traits of Snef3255 and provide clues for mining key functional genes.

Secreted proteins play a vital role in the process of *Trichoderma*-induced plant resistance. Among them, Cerato-platanin family proteins and LysM domain-containing proteins are important, secreted proteins confirmed to participate in *Trichoderma*-plant interactions [26,27,98]. Previous studies have shown that CP family proteins such as Sm1/Epl1 can induce systemic resistance in plants and enhance defense against pathogens and nematodes [26,105]; LysM proteins can regulate recognition between fungi and plants and the activation of immune responses by binding to chitin oligomers [27]. To date, most studies have focused on identifying and functionally validating secreted proteins, while relatively little is known about their interacting target proteins in host plants, particularly in peanut. In this study, two secreted proteins of Snef3255 (ThCP1 and ThLysM1) were identified. The interacting proteins of these two effectors in peanut were screened via Y2H assays, and the authenticity of these protein–protein interactions was validated by Co-IP and BiFC assays. These findings clarify the potential molecular pathways through which Snef3255 regulates peanut immune responses. These preliminary results provide candidate molecular clues for further investigating secreted protein-mediated interactions among Snef3255, peanut and RKNs. In addition to the identified secreted proteins, other secondary metabolism-related genes, including those encoding PKSs, NRPSs, and cytochrome P450 enzymes, may also warrant further investigation, although their functions remain to be experimentally determined.

## 5. Conclusions

In conclusion, *T. harzianum* Snef3255 exhibited excellent nematicidal activity against peanut RKN disease and significant yield-promoting effects, making it a promising biocontrol strain. The high-quality chromosome-level genome assembly, together with abundant biocontrol-related genes, indicated that multiple genes jointly endow it with biocontrol capabilities. The identification of candidate host proteins that interact with ThCP1 and ThLysM1, although still preliminary and requiring further experimental validation, provided a valuable starting point for elucidating the molecular mechanisms underlying *Trichoderma*–peanut interactions. Collectively, these findings provided valuable strain resources and a theoretical foundation for the sustainable management of peanut RKN disease.

## Figures and Tables

**Table 1:** Effects of different treatments on nematode population density, disease index, biocontrol efficacy and yield of peanut in pot experiments.

	Gall Index	Efficacy (%)	Egg Masses/g Root	Eggs/g Root	Number of Females/g Root	J2s/200 mL Soil	Yield (g)	Percent Yield Increase (%)
CK	74.67 ± 1.63 a	/	22.1 ± 0.64 a	703.9 ± 3.44 a	23.83 ± 0.56 a	416.47 ± 1.24 a	34.6 ± 0.09 d	/
TC	20 ± 1.26 d	73.22 ± 1.69 b	6.27 ± 0.27 d	191.87 ± 0.84 d	7.03 ± 0.15 d	124.93 ± 1.76 d	41.04 ± 0.13 a	18.61 ± 0.37 a
TS	24.67 ± 1.03 c	66.97 ± 1.38 c	7.13 ± 0.21 c	224.7 ± 0.88 c	8.4 ± 0.52 c	149.75 ± 0.75 c	39.42 ± 0.32 b	13.92 ± 0.92 b
CH1	16.33 ± 1.51 e	78.57 ± 1.69 a	4.43 ± 0.39 e	141.3 ± 0.37 e	5.6 ± 0.44 e	97.3 ± 0.62 e	41.26 ± 1.06 a	19.23 ± 3.06 a
CH2	28.67 ± 1.03 b	61.61 ± 1.38 d	8.23 ± 0.37 b	247.97 ± 1.23 b	9.37 ± 0.29 b	169.87 ± 1.22 b	37.37 ± 0.3 c	8.01 ± 0.85 c

Different lowercase letters denote significant differences as determined by Duncan’s multiple range test (*p* < 0.05).

**Table 2:** Effects of different treatments on nematode population density, disease index, biocontrol efficacy and yield of peanut in field experiments.

	Gall Index	Efficacy (%)	Egg Masses/g Root	Eggs/g Root	Number of Females/g Root	J2s/200 mL Soil	Yield (g)	Percent Yield Increase (%)
CK	74.33 ± 1.97 a	/	21.7 ± 0.21 a	650.8 ± 1.26 a	23.37 ± 0.32 a	356.77 ± 1.34 a	34.47 ± 2.92 d	/
TC	21.67 ± 1.51 d	70.85 ± 2.02 b	5.43 ± 0.15 d	186.63 ± 0.43 d	6.43 ± 0.23 d	110.1 ± 1.27 d	40.73 ± 1.06 a	18.15 ± 3.08 a
TS	28.33 ± 0.82 c	61.88 ± 1.1 c	6.53 ± 0.16 c	215.77 ± 0.9 c	7.6 ± 0.13 c	128.7 ± 1.05 c	39.16 ± 0.2 b	13.61 ± 0.6 b
CH1	17.67 ± 0.82 e	76.23 ± 1.1 a	4.07 ± 0.16 e	173.63 ± 1.08 e	4.8 ± 0.28 e	88.57 ± 1.2 e	41.03 ± 1.06 a	19.02 ± 3.08 a
CH2	33 ± 1.1 b	55.61 ± 1.47 d	7.47 ± 0.33 b	249.6 ± 1.21 b	8.87 ± 0.33 b	146.83 ± 1.49 b	37.21 ± 0.33 c	7.95 ± 0.94 c

Different lowercase letters denote significant differences as determined by Duncan’s multiple range test (*p* < 0.05).

**Table 3:** Assembly statistics and genomic features of Snef3255.

Assembly Feature	*T. harzianum* Snef3255
Genome size (bp)	40,811,729
Sequence number	7
N50 (bp)	6,280,179
GC content (%)	48.28
Short-read mapping (%)	99.06
Short-read coverage (%)	99.99
BUSCO (%)	99.31
Chromosome size (bp)	
Chromosome 1	7,924,814
Chromosome 2	6,931,035
Chromosome 3	6,229,813
Chromosome 4	6,280,179
Chromosome 5	5,414,076
Chromosome 6	4,721,481
Chromosome 7	3,310,331

**Table 4:** General genomic features and functional annotation of Snef3255.

Type	*T. harzianum* Snef3255
Gene number	13,611
Gene length (bp)	26,533,389
Average gene length (bp)	1949.41
Exon number	39,296
Exon length (bp)	23,919,909
Average exon length (bp)	608.71
Average exon per gene	2.89
CDS number	38,538
CDS length (bp)	19,002,006
Average CDS length (bp)	493.07
Average CDS per gene	2.83
Pesudogene number	31
Pesudogene size (bp)	74,198
rRNA number	74
tRNA number	223
other ncRNA number	35
Gene annotation number	12,096
nr	12,086
TrEMBL	12,088
Pfam	9106
Swissprot	7360
GO	8352
KEGG	3379
KOG	6099
CAZyme	627
AA	91
CBM	98
CE	118
GH	299
GT	100
PL	6
TCDB	136
PHI	3929
CYPED	521
antiSMASH	53

**Table 5:** Unique gene clusters of Snef3255.

Region	Swiss-Prot Hit	GO Annotation	KEGG Annotation	Gene Cluster
Region 6.3	Q4WTT9	GO:0042493 cellular response to drug	ko00980 Metabolism of xenobiotics by cytochrome P450	uncharacterized gene cluster
Region 2.7	A2EYC4	GO:0009072 aromatic amino acid family metabolic process	ko01110 Biosynthesis of secondary metabolites	PKS biosynthetic gene cluster

Regions predicted by antiSMASH based on annotated genes.

**Table 6:** Conserved gene clusters and gene families of *Trichoderma* species.

Regions	Swiss-Prot Hit	GO Annotation	KEGG Annotation	Gene Cluster
Region 7.1	P46828	GO:0019184 nonribosomal peptide biosynthetic processZFIN	ko01053 Nonribosomal peptide structures biosynthesis	NRPS biosynthetic gene cluster
Region 7.2	A0A0S2E3G7	GO:0042282 hydroxymethylglutaryl-CoA reductase activity	ko00380 Tryptophan metabolism	6-PP PKS1 core biosynthetic gene cluster

Regions predicted by antiSMASH based on annotated genes.

## Data Availability

The whole-genome assembly is available under the accession number GWHISUI00000000.1 (BioProject ID: PRJCA062073; BioSample ID: SAMC7475426). The chromosomal sequences from Chr1 to Chr7 are assigned the accession numbers ranging from GWHISUI00000001.1 to GWHISUI00000007.1. Additionally, the ITS and *RPB2* gene sequences were deposited with the accession numbers C_AA219805.1 and C_AA168267.1, respectively. Both datasets are publicly accessible at https://ngdc.cncb.ac.cn (accessed on 14 August 2026).

## Supplementary Materials

The following supporting information can be downloaded at: https://www.mdpi.com/article/10.3390/microorganisms14081851/s1, Table S1: The primers used in this research; Table S2: Thermal cycling parameters for PCR amplification; Table S3: Grading criteria for root-knot nematode disease severity.

## Abbreviations

The following abbreviations are used in this manuscript:

6-PP	6-pentyl-α-pyrone
ANI	Average Nucleotide Identity
BUSCO	Benchmarking Universal Single-Copy Orthologs
CAZy	Carbohydrate-Active enZYmes
CDD	Conserved Domain Database
CFU	Colony-forming unit
CYPED	Cytochrome P450 Engineering Database
Hi-C	High-throughput chromatin conformation capture
ITS	Internal transcribed spacer
J2s	Second-stage juveniles
KEGG	Kyoto Encyclopedia of Genes and Genomes
KOG	Eukaryotic Orthologous Groups
MAMPs	Microbe-associated molecular patterns
NCBI	National Center for Biotechnology Information
NRPS	Non-ribosomal peptide synthetase
ONT	Oxford Nanopore Technologies
PDA	Potato dextrose agar
PDB	Potato dextrose broth
PHI	Pathogen-Host Interaction database
PKS	Polyketide synthase
*RPB2*	RNA polymerase II second largest subunit gene
RKN	Root-knot nematode
RKNs	Root-knot nematodes
TCDB	Transporter Classification Database
TFB	*Trichoderma* fermentation broth treatment
TSC	*Trichoderma* seed coating treatment
Y2H	Yeast two-hybrid
