# Supplementary material for: Isolation, Biocontrol Efficacy and Genomic Analysis of Trichoderma harzianum Snef3255 Against Peanut Root-Knot Nematode Disease
Source: Microorganisms. 2026 Aug 20;14(8):1851. doi: 10.3390/microorganisms14081851 (PMC13515719; doi:10.3390/microorganisms14081851)
Supplement: Supplementary file 1 [file microorganisms-14-01851-s001.zip › Table S1.pdf]

Table S1. The primers used in this research.

Primer name	Primer sequence
ITS1	TCCGTAGGTGAACCTGCGG
ITS4	TCCTCCGCTTATTGATATGC
fRPB2-F	GAYGAYMWGATGCAYYTTYGG
fRPB2-R	CCRARWTCKRTCRATG
clone-*EF-F*	CATCGAGAAGTTCGAGAAGG
clone-*EF-R*	AACTTGCAGGCAATGTGG
clone-*Thcp1*-F	GGACCTGCATATGGCATGCAATTGTCCAGCCTCTTCA
clone-*Thcp1*-R	CGCTGCAGGTCGACGTTAAAGACCGCAGTTCTTGACA
clone-*LysM*-F	GGACCTGCATATGGCATGAGACAGTCAATTGCTGTTG
clone-*LysM*-R	CGCTGCAGGTCGACGTCATATGAGGCCGATGCAAACA
clone-*SRC2*-F	GCCATGGAGGCCAGTGAATTCATTAAGTTCCACTTCTCCCTGGTTC
clone-*SRC2*-R	ACGATTCATCTGCAGCTCGAGTAGAGCAAAATTTAAATATTTCAAGA
clone-*PR1*-F	GCCATGGAGGCCAGTGAATTCCAATATTGTCATCAAACCCCTTAATAT
clone-*PR1*-R	ACGATTCATCTGCAGCTCGAGTTAAAATAAAATATAACAAACATCATTATTAT
clone-*CP*-F	GCCATGGAGGCCAGTGAATTCATGGCTGAAACCCGTAACGC
clone-*CP*-R	ACGATTCATCTGCAGCTCGAGAAACCATCATCAGAAGCATATATATAAGAG
clone-*LysM*-F	GCCATGGAGGCCAGTGAATTCATGTGCCATTGTGAACTATAGAAGGT
clone-*LysM*-R	ACGATTCATCTGCAGCTCGAGTTACACATAAAACACATTAATATTTCACTCTC
clone-PR*1*-C-F	GTCGACATGGTCGACGGTATCATGCAATTGTCCAGCCTCTTC
clone-PR*1*-C-R	ACTAGTTCTAGAGCGGCCGCACAAGACCGCAGTTCTTGAC
clone-SRC2-F	GTCGACATGGTCGACGGTATCATGAGACAGTCAATTGCTGTTGTG
clone-SRC2-F	ACTAGTTCTAGAGCGGCCGCCATATGAGGCCGATGCAAACAT
clone-*cp*-B-F	GTCGACATGGTCGACGGTATCATGCAATTGTCCAGCCTCTTC
clone-*cp*-B-R	ACTAGTTCTAGAGCGGCCGCACAAGACCGCAGTTCTTGAC
clone-*LysM*-B-F	GTCGACATGGTCGACGGTATCTGAGGCCGA
clone-*LysM*-B-R	ACTAGTTCTAGAGCGGCCGCTGTGCCATTG
clone-*PR1*-B-F	GTCGACATGGTCGACGGTATCAAATAAAATATAACAAACATC
clone-*PR1*-B-R	ACTAGTTCTAGAGCGGCCGCATAAAACACATTAATACGA
clone-*SRC2*-F	GTCGACATGGTCGACGGTATCATTGTCCAGCCTCTTCA
clone-*SRC2*-R	ACTAGTTCTAGAGCGGCCGCTTAAAGACCGCAGTTCTTGACA

`
