# Supplementary material for: Isolation, Biocontrol Efficacy and Genomic Analysis of Trichoderma harzianum Snef3255 Against Peanut Root-Knot Nematode Disease
Source: Microorganisms. 2026 Aug 20;14(8):1851. doi: 10.3390/microorganisms14081851 (PMC13515719; doi:10.3390/microorganisms14081851)
Supplement: Supplementary file 1 [file microorganisms-14-01851-s001.zip › Table S2.pdf]

Table S2. Thermal cycling parameters for PCR amplification.

**Step Type**	**Temperature (°C)**	**Duration (min)**	**Cycle Number**
Hot Start Activation	95	5	1
Denaturation	95	1	30
Annealing	55	2	30
Extension	72	2	30
Final Extension	72	10	1

`
