# Supplementary material for: Isolation, Biocontrol Efficacy and Genomic Analysis of Trichoderma harzianum Snef3255 Against Peanut Root-Knot Nematode Disease
Source: Microorganisms. 2026 Aug 20;14(8):1851. doi: 10.3390/microorganisms14081851 (PMC13515719; doi:10.3390/microorganisms14081851)
Supplement: Supplementary file 1 [file microorganisms-14-01851-s001.zip › Table S3.pdf]

Table S3. Grading criteria for root-knot nematode disease severity.

Grade	Description of root-knot nematode disease severity
0	No root knots on the root system
1	A small number of tiny root knots, almost undetectable to the naked eye
2	A large number of small root knots, yet still inconspicuous
3	Some visible large root knots
4	Predominantly large root knots, with the taproot remaining clean
5	Root knots present on the taproot, approximately 50% of the root system infected
6	Root knots on most taproots, with a reduced root system
7	Root knots on all taproots
8	Severe root knot formation on all roots, almost no healthy roots remaining
9	Severe root knot formation on all roots, plants usually moribund
10	Root system almost completely destroyed, plants usually dead

`
